# Site-Specific Bioconjugation of an Organometallic Electron Mediator to an Enzyme with Retained Photocatalytic Cofactor Regenerating Capacity and Enzymatic Activity

**DOI:** 10.3390/molecules20045975

**Published:** 2015-04-07

**Authors:** Sung In Lim, Sungho Yoon, Yong Hwan Kim, Inchan Kwon

**Affiliations:** 1 Department of Chemical Engineering, University of Virginia, Charlottesville, VA 22904, USA; E-Mail: sl2nc@virginia.edu; 2 Department of Bio & Nano Chemistry, Kookmin University, 861-1 Jeoungnung-dong, Seongbuk-gu, Seoul 136-702, Korea; E-Mail: yoona@kookmin.ac.kr; 3 Department of Chemical Engineering, Kwangwoon University, Seoul 139-701, Korea; E-Mail: metalkim@kw.ac.kr; 4 School of Materials Science and Engineering, Gwangju Institute of Science and Technology (GIST), Gwangju 500-712, Korea

**Keywords:** electron mediator, non-natural amino acid, redox enzyme, site-specific bioconjugation, formate dehydrogenase

## Abstract

Photosynthesis consists of a series of reactions catalyzed by redox enzymes to synthesize carbohydrates using solar energy. In order to take the advantage of solar energy, many researchers have investigated artificial photosynthesis systems mimicking the natural photosynthetic enzymatic redox reactions. These redox reactions usually require cofactors, which due to their high cost become a key issue when constructing an artificial photosynthesis system. Combining a photosensitizer and an Rh-based electron mediator (RhM) has been shown to photocatalytically regenerate cofactors. However, maintaining the high concentration of cofactors available for efficient enzymatic reactions requires a high concentration of the expensive RhM; making this process cost prohibitive. We hypothesized that conjugation of an electron mediator to a redox enzyme will reduce the amount of electron mediators necessary for efficient enzymatic reactions. This is due to photocatalytically regenerated NAD(P)H being readily available to a redox enzyme, when the local NAD(P)H concentration near the enzyme becomes higher. However, conventional random conjugation of RhM to a redox enzyme will likely lead to a substantial loss of cofactor regenerating capacity and enzymatic activity. In order to avoid this issue, we investigated whether bioconjugation of RhM to a permissive site of a redox enzyme retains cofactor regenerating capacity and enzymatic activity. As a model system, a RhM was conjugated to a redox enzyme, formate dehydrogenase obtained from *Thiobacillus sp*. KNK65MA (TsFDH). A RhM-containing azide group was site-specifically conjugated to *p*-azidophenylalanine introduced to a permissive site of TsFDH via a bioorthogonal strain-promoted azide-alkyne cycloaddition and an appropriate linker. The TsFDH-RhM conjugate exhibited retained cofactor regenerating capacity and enzymatic activity.

## 1. Introduction

Plants use solar energy to synthesize glucose as a chemical energy reserve, in a process called photosynthesis. Chemically, photosynthesis is a series of redox reactions. Electrons donated by water are excited by light and, through photosystem I and II, stored in energy-rich ATP and NAD(P)H cofactors. Reduction of carbon dioxide to carbohydrates is catalyzed by redox enzymes fueled by cofactors. Photosynthesis is an elegant example of how Nature exploits the most abundant renewable energy. Efforts have been made to construct artificial photosynthesis systems mimicking natural photosynthesis to synthesize fine chemicals [1,2]. They consist of a photocatalytic cofactor regeneration step coupled to a biocatalytic CO_2_ fixation via a redox enzyme. As artificial counterparts to the natural photosystem I and II, various organic or inorganic photosensitizers have been developed to photo-excite electrons provided by sacrificial donors such as triethanolamine (TEOA) and ethylenediaminetetraacetic acid (EDTA) [3,4]. Electron mediators receive high-energy electrons from photosensitizers and work as an electron shuttle to (re)generate biologically active cofactors.

Cofactor regeneration efficiency, which governs the flow of the entire system, depends critically on the specificity of an electron mediator. The key issue is the capacity to minimize the formation of NAD(P)H isomers that are not recognized as a cofactor by redox enzymes while regioselectively generating bioactive 1,4-NAD(P)H. A rhodium (Rh)-coordinated organometallic electron mediator, [Rh(bpy)(Cp*)H_2_O]^2+^ where bpy is bipyridine and Cp* is pentamethylcyclopentadienyl, has been the most popular organometallic electron mediator due to its stability and specificity [5,6]. However, it suffers from cost-inefficiency. Regenerated NAD(P)H can be utilized by a redox enzyme, and then oxidized cofactors can be recycled back to NAD(P)H by photocatalysis. Therefore, photocatalytic cofactor regeneration system combined with redox enzyme has been used to achieve artficial photosynthesis [7,8,9,10]. Since electron mediators are expensive, it is not economical to maintain the high concentration of electron mediator required for efficient enzymatic reactions using high concentrations of NAD(P)H. We hypothesized that conjugation of an electron mediator to a redox enzyme would reduce the amount of electron mediators required for efficient enzymatic reactions. Because NAD(P)Hs are photocatalytically regenerated, they are readily available to a redox enzyme, therefore the local NAD(P)H concentration near an enzyme becomes higher. In particular, when mixing enzymatic reaction components is not allowed, such as inside a small portable device, the higher local NAD(P)H concentration results in a substantially higher enzymatic reaction rate.

Conventional conjugation of an organic molecule to amino or thiol groups of enzymes [11,12] often results in substantial loss of enzymatic activity. This is due to conjugation occurring at non-permissive enzyme sites. We have demonstrated that site-specific bioconjugation to a fluorescent protein or enzyme can be achieved using site-specific incorporation of a non-natural amino acid containing a bioorthogonal functional group, resulting in almost fully-retained activities upon conjugation of fatty acid or biotin, respectively [13,14]. Similarly, we hypothesized that conjugation of electron mediator to a permissive site of a redox enzyme will result in a conjugate with retained enzymatic activity and cofactor regeneration capacity. We also hypothesized that such a site-specific conjugation of an organometallic electron mediator will circumvent the mutual inactivation of the electron mediator and redox enzyme [15,16]. Although the number of electron mediators to be conjugated and conjugation sites should be optimized, conjugation of the electron mediator to specific sites will reduce the mutual inactivation often caused by electron mediator binding to catalytically critical sites.

As a model redox enzyme, we investigated formate dehydrogenase obtained from *Thiobacillus sp*. KNK65MA (TsFDH). Formate dehydrogense belongs to the superfamily of D-specific 2-hydroxyacid dehydrogenases and catalyzes the oxidation of formate to CO_2_. Although it is well known as a standard enzyme for many enzymatic NADH regeneration reactions using formate as a substrate, its unique reverse reaction (CO_2_ reduction to formate) is beneficial in building a biocatalytic CO_2_ fixation system due to its specific activity [8]. Therefore, we tethered TsFDH and (Rh)-coordinated organometallic electron mediator (RhM) to retain significant catalytic activity of TsFDH with the ultimate goal of constructing an efficient artificial photosynthesis system. In this study, we assumed that the operational stability of TsFDH is higher than, or at least comparable, to that of RhM. Then, the use of TsFDH-RhM conjugate is less costly than the use of TsFDH and RhM separately. However, if not, the regeneration of the Rh would be an alternative option to reduce the cost of cofactor regeneration [17].

## 2. Results and Discussion

### 2.1. Site-Specific Incorporation of p-Azido-L-Phenylalanine (pAzF) into Formate Dehydrogenase

**Figure 1:**
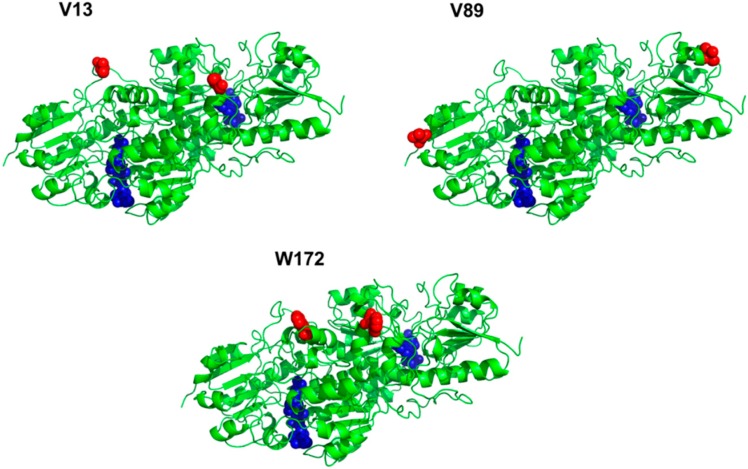
Selection of pAzF incorporation sites in the homodimeric TsFDH. Incorporation site (red sphere); active site (blue sphere).

The artificial photosynthesis system comprises TEOA (electron donor), eosin Y (dye photosensitizer), NAD+ (cofactor), RhM-azide (azide-functionalized Rh-based electron mediator), and formate dehydrogenase obtained from *Thiobacillus sp*. KNK65MA (TsFDH). TsFDH is expected to reduce CO_2_ to formate using NADH regenerated by photocatalytic reaction *in situ* as well as its natural function of converting formate into CO_2_ [18]. As described in the Introduction, we focused on conjugating RhM-azide to TsFDH to facilitate the delivery of NADH regenerated to TsFDH. In order to conjugate RhM-azide to a specific site of TsFDH via strain-promoted azide-alkyne cycloaddition (SPAAC), site-specific incorporation of pAzF into TsFDH was performed. Optimal pAzF incorporation sites were determined based on solvent accessibility, hydrophobicity, and spatial orientation relative to the NADH-binding pocket of TsFDH (active site). First, hydropathy index and three-dimensional structure analyses were added to narrow the choices. Considering the hydrophobic nature of pAzF, only hydrophobic amino acids were evaluated. Then, only amino acids not directly involved in the function of TsFDH were considered based on the three-dimensional structure analysis, leading to three candidate sites (V13, V89, and W172) (Figure 1). Finally, the solvent accessibility of each amino acid consisting of TsFDH was analyzed by ASA view, a solvent accessibility calculation program of a protein [19] using the crystal structure of TsFDH [20]. The solvent accessibility of each site is summarized in Table 1. Since V13 site has the highest solvent accessibility among three sites, we chose V13 as a pAzF incorporation site.

**Table 1:** Solvent accessibility of three incorporation sites in the TsFDH.

Position	V13	V89	W172
**Solvent accessibility**	0.70	0.38	0.32

**Figure 2:**
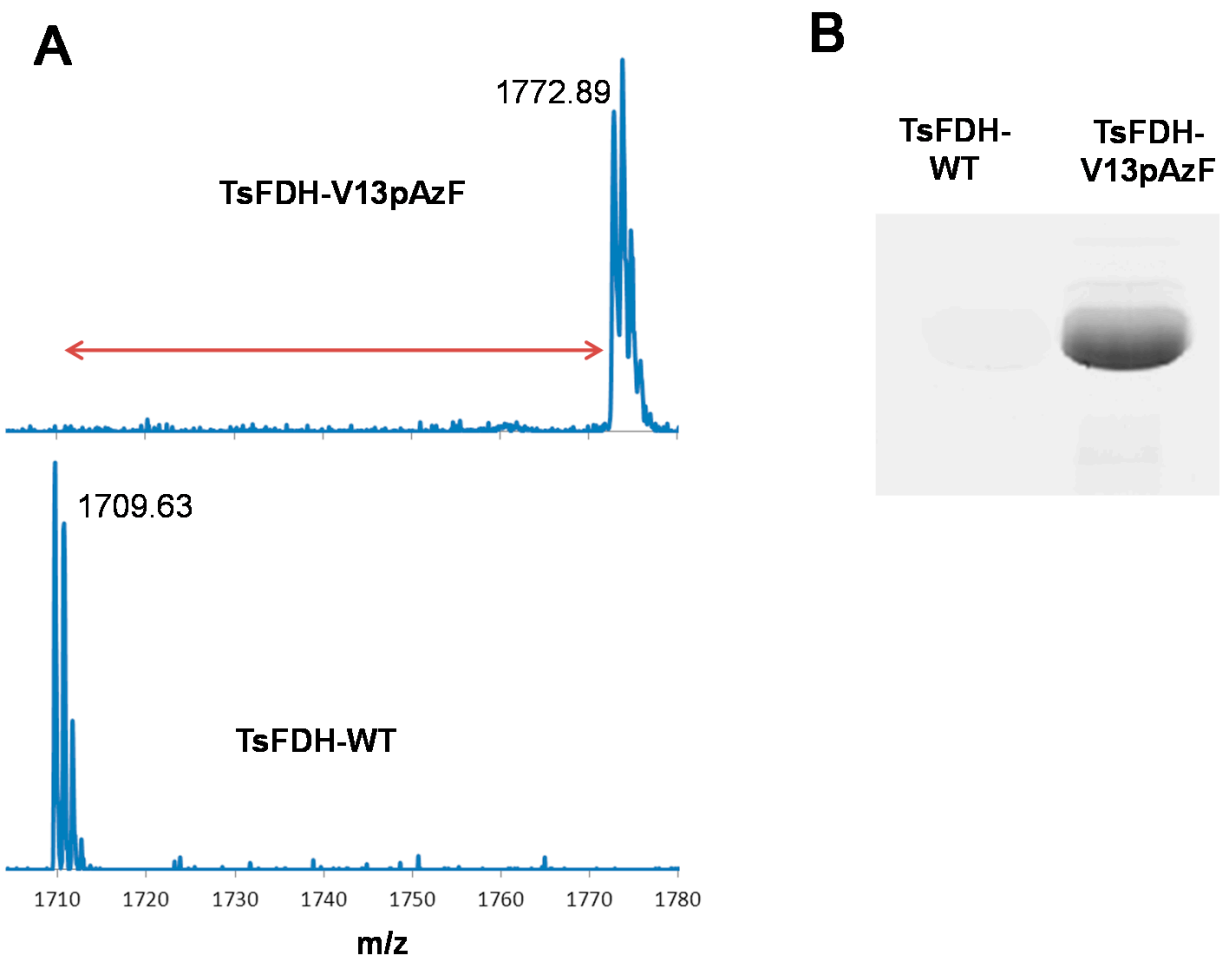
(**A**) Verification of pAzF incorporation into TsFDH by MALDI-TOF MS; mass spectra of one tryptic digest of TsFDH-V13pAzF (residues 4-18; ILCVLYDDP *pAzF*DGYPK) (*top*) and TsFDH-WT (residues 4-18; ILCVLYDDPVDGYPK) (*bottom*) and (**B**) in-gel fluorescence of TsFDH-WT and TsFDH-V13pAzF obtained by the SPAAC-mediated DBCO-dye labeling.

The TsFDH-V13pAzF was produced from *E. coli* C321ΔA.exp cells transformed with pQE-80 TsFDH-V13Amb plasmid in LB media supplemented with pAzF and other components as described in the Experimental Section. The produced yield of purified TsFDH-V13pAzF was 16 mg/L in a culture volume basis. Incorporation of pAzF and its reactivity towards SPAAC was verified by MALDI-TOF mass spectrometry (MS) and SPAAC dye labeling, respectively. For TsFDH-WT, one tryptic digest (residues 4-18; ILCVLYDDPVDGYPK) containing V13 was found at *m/z* 1,709.63 (theoretical *m/z* = 1,709.84) in the mass spectrum (Figure 2A, bottom). The replacement of V13 (117.15 Da) with pAzF (206.20 Da) was expected to increase the mass by +89.05 a.m.u. For TsFDH-V13pAzF, one tryptic digest (Residues 4-18; ILCVLYDDP*pAzF*DGYPK) was found at *m/z* 1,772.89 (Figure 2A, top), which corresponds to the mass shift by +63.26 a.m.u. compared to that of TsFDH-WT. The discrepancy between the expected and measured mass can be attributed to the fragmentation of pAzF upon irradiation. It was previously shown that an aryl azide can be fragmented during matrix-assisted laser desorption ionization leading to the generation of amine group and decrease in mass by about 26 Da [21,22,23,24].

To verify the incorporation of pAzF into TsFDH variant, labeling of TsFDH variants using a fluorescent dye containing an azide-reactive dibenzocyclooctyne (DBCO) group was performed. TsFDH-V13pAzF and TsFDH-WT were reacted with a DBCO-PEG_4_-carboxyrhodamine (DBCO-dye), respectively. The reaction mixtures were analyzed using in-gel fluorescence analysis. TsFDH-V13pAzF exhibited high fluorescence upon excitation, whereas TsFDH-WT did not exhibit any detectable fluorescence (Figure 2B). Combined results of MALDI-TOF MS and in-gel fluorescence analysis using DBCO-dye labeling clearly indicated that pAzF was incorporated into the V13 site of TsFDH and was reactive towards SPAAC.

### 2.2. Conjugation of Rh-Based Electron Mediator to TsFDH-V13pAzF

**Figure 3:**
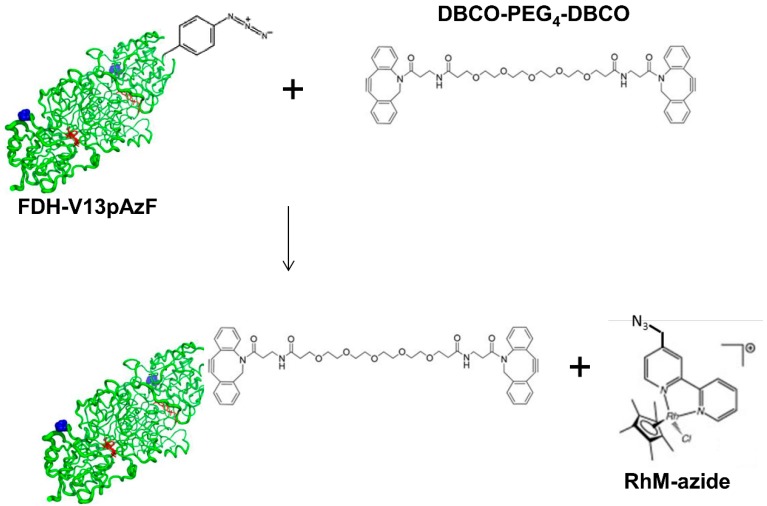
Schematic representation of conjugation of RhM-azide to TsFDH-V13pAzF via DBCO-PEG_4_-DBCO bifunctional linker through SPAAC. Not drawn to scale.

**Figure 4:**
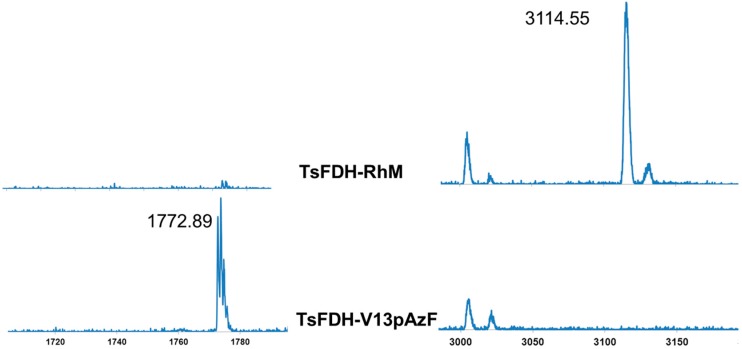
Verification of the RhM conjugation by MALDI-TOF MS. Spectra of TsFDH-RhM (**Top row**) and TsFDH-V13pAzF (**Bottom row**). See the main text for more details.

Next, we performed site-specific conjugation of RhM-azide to TsFDH-V13pAzF using a bifunctional linker containing two dibenzocyclooctyne (DBCO) groups connected by a short polyethyleneglycol linker (PEG_4_) (Figure 3). In the first step, TsFDH-V13pAzF was mixed with the bifunctional linker to generate a TsFDH-DBCO-PEG_4_-DBCO intermediate, and then with RhM-azide which contains an azide functionality in one pyridine ring. Success of the RhM-azide conjugation was confirmed by MALDI-TOF MS (Figure 4). When compared to the tryptic digest of TsFDH-V13pAzF (residues 4-18; ILCVLYDDP*pAzF*DGYPK), the original peak representing the pAzF incorporation, *m/z* 1772.89, was not detected, while a new peak was observed at *m/z* 3114.55. The new peak indicated that there was an increase in the mass of the tryptic digest of TsFDH-V13pAzF (without fragmentation: *m/z* 1798.85) by conjugation of RhM-azide (without any chloride ion; 462.16 Da) through the bi-functional linker (approximately 855 Da).

### 2.3. Characterization of the TsFDH-RhM Conjugate: Enzymatic Activity and Cofactor Generation

**Figure 5:**
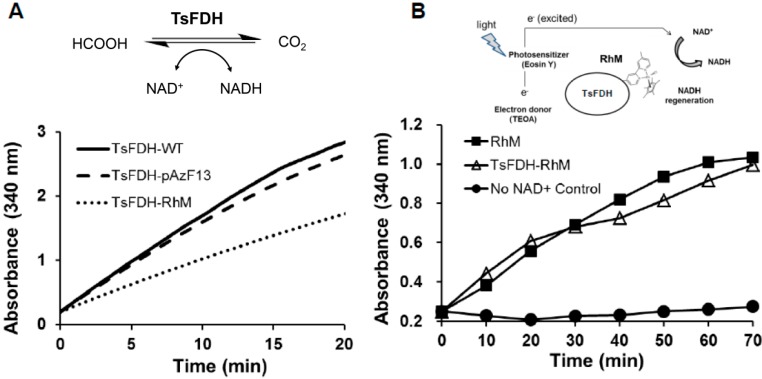
Characterization of TsFDH-RhM. (**A**) NAD^+^-dependent conversion of formate to carbon dioxide by TsFDH-RhM (40 nM), TsFDH-V13pAzF (40 nM), TsFDH-WT (40 nM), and RhM (40 nM); (**B**) Photo-induced generation of NADH. The reaction mixture consisting of TEOA (5%), eosin Y (20 µM), NAD^+^ (0.8 mM), and TsFDH-RhM (40 μM) or free RhM (40 μM) was illuminated by white light at 37 °C.

To investigate the effect of RhM conjugation as well as pAzF incorporation on native activity of TsFDH, NAD^+^-dependent formate oxidation by TsFDH-RhM (40 nM), TsFDH-V13pAzF (40 nM), TsFDH-WT (40 nM), and RhM alone (40 nM) was measured in parallel by monitoring conversion of NAD^+^ into NADH at 340 nm (Figure 5A). Relative to TsFDH-WT, TsFDH-V13pAzF had 92% enzymatic activity. However, after RhM conjugation, the activity was reduced to 60% via non-specific inhibition. This was probably the result of distorted folded structure or modification of critical residues in TsFDH by reactive rhodium metals in RhM [5]. Electron-mediating function of the conjugated RhM was also measured by subjecting the TsFDH-RhM conjugate to the photo-induced NADH regeneration system under the conditions reported previously with some modifications in the reagent concentrations [25,26] (Figure 5B). A photosensitizer eosin Y photo-excites electrons obtained from a sacrificial donor TEOA. The electron mediator RhM conjugated to TsFDH was expected to deliver the high-energy electrons from the photosensitizer to NAD^+^ resulting in NADH formation. The TsFDH-RhM performed as effectively as a standard system consisting of free (unconjugated) RhM at the same concentration. These results indicate that the TsFDH-RhM retained both enzymatic and photocatalytic activities although the former underwent 40% reduction. To our knowledge, this is the first study demonstrating the feasibility of site-specific conjugation of a metallic electron mediator to formate dehydrogenase while retaining enzymatic and photocatalytic activities.

## 3. Experimental Section

### 3.1. General Information

*p*-Azido-L-phenylalanine (pAzF) was obtained from Chem-Impex International (Wood Dale, IL, USA). Ni-nitrilotriacetic acid (NTA) agarose and pQE80 plasmid were purchased from Qiagen (Valencia, CA, USA). DBCO-PEG_4_-carboxyrhodamine and DBCO-PEG_4_-DBCO were purchased from Bioconjugate Technology Company (Scottsdale, AZ, USA). ZipTip C18 was obtained from Millipore Corporation (Billerica, MA, USA). Sequencing grade-modified trypsin was obtained from Promega Corporation (Madison, WI, USA). PD-10 desalting columns were purchased from GE Health care (Piscataway, NJ, USA). All chemicals were obtained from Sigma-Aldrich Corporation (St. Louis, MO, USA) unless otherwise stated. ^1^H-NMR spectra were recorded on Varian 500 MHz instruments. The SynergyTM four multimode microplate reader (BioTek, Winooski, VT, USA) was used to monitor spectrometric changes. The Biospectrum imaging system (UVP Inc., Upland, CA, USA) was used to visualize fluorescently labeled proteins. The portable white light illuminator (Promark International Inc., Bartlett, IL, USA) was used to induce the photocatalytic cofactor regeneration.

### 3.2. Synthesis of [Cp*Rh(4-(Azidomethyl)-4'-Methyl-2,2'-Bipyridine)Cl]Cl (**RhM-azide**)

**Scheme 1:**
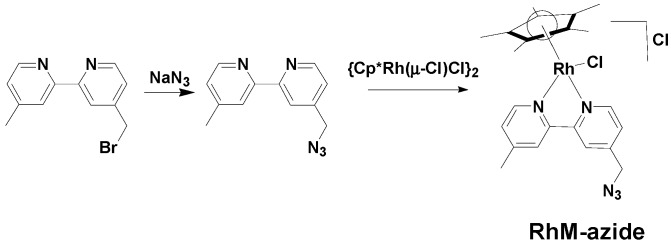
Synthesis scheme of RhM-azide.

A scheme of the synthesis route to RhM-azide is shown in Scheme 1. NaN_3_ (0.032 g, 0.496 mmol) and dimethylsulfoxide (DMSO, 7 mL) were taken in a 50 mL round bottom flask and stirred for 12 h at ambient temperature. A DMSO solution (8 mL) of 4-(bromomethyl)-4'-methyl-2,2'-bipyridine (0.100 g, 0.381 mmol) was added and the stirring continued for 10 h. The resulting light brown solution was diluted with ethyl acetate (20 mL) and water (10 mL). The organic layer was separated, dried over Na_2_SO_4_ and concentrated under reduced pressure, affording 4-(azidomethyl)-4'-methyl-2,2'-bipyridine (0.070 g, yield: 82%) as a light brown liquid, {η^5^-Cp^*^Rh(μ-Cl)Cl}_2_ (0.061 g, 0.100 mmol) was added to a solution of the azide ligand (0.045 g, 0.200 mmol) in methanol (6 mL), and stirred for 6 h at ambient temperature under a nitrogen atmosphere. Upon reduction of the solvent volume to 1 mL and sequential addition of 1 mL diethyl ether, a yellow precipitate was generated. The precipitate were filtered and dried under reduced pressure (0.085 g, Yield = 79%). ^1^H-NMR (500 MHz, CD_3_OD) δ 8.95 (*d*, *J* = 5.8 Hz, 1H), 8.82 (*d*, *J* = 5.7 Hz, 1H), 8.52 (*s*, 1H), 8.47(*s*, 1H), 7.84 (*dd*, *J* = 5.7, 1.0 Hz, 1H), 7.71 (*dd*, *J* = 5.7, 1.0 Hz, 1H), 4.83 (*s*, 2H), 2.64 (*s*, 3H), 1.72 (*s*, 15H).

### 3.3. Site-Specific Incorporation of pAzF into Formate Dehydrogenase

The *fdh* gene, originally obtained from *Thiobacillus sp.* KNK65MA, with an additional C-terminal histidine sequence, was PCR amplified by a pair of primers, 5'-TTCACACAGAATTCATTAAAGAGGAGAAATTAACTATGGCCAAAATCCTGTGCG-3' and 5'-GCTAATTAAGCTTAGTGATGGTGATGGTGATGACCCGCTTTTTTGAATTTTGCG-3', using pET-23b(+) plasmid containing the *fdh* gene [27] as a template, and subcloned into pQE-80 plasmid by the restriction-free cloning method [28] to generate pQE-80 TsFDH plasmid. A polymerase chain reaction (PCR)-based amber mutation was employed to generate pQE-80 TsFDH-V13Amb. Then, pQE-80 TsFDH-V13Amb was transformed into RF1-free *E. coli* strain, C321ΔA.exp [29] to generate C321ΔA.exp (pQE-80 TsFDH-V13Amb).

The seed culture of C321ΔA.exp (pQE-80 TsFDH-V13Amb) was transferred into fresh 2×YT medium (100 µg/mL ampicillin and 35 µg/mL chloramphenicol) and incubated at 220 rpm at 37 °C. When the OD_600_ of cell culture reached 0.5, pAzF solution was added to a final concentration of 1 mM. After 10 min, 1 mM isopropyl β-D-1-thiogalactopyranoside (IPTG) and 0.2% (w/v) L-(+)-arabinose were added to the cell culture in order to induce TsFDH expression. At 5 h post-induction, cells were harvested, and pelleted by centrifugation at 5,000 rpm for 10 min followed by storage at −20 °C.

TsFDH variant was purified using metal-affinity chromatography according to the manufacturer’s protocol (Qiagen), and then buffer-exchanged to 50 mM 4-(2-hydroxyethyl)-1-piperazine-ethanesulfonic acid (HEPES)/0.2 M NaCl buffered at pH 8.2 using a PD-10 column. Expression of wild-type TsFDH (TsFDH-WT) was performed using TOP10 cells transformed with pQE-80 TsFDH plasmid with 1 mM IPTG induction. Purification of TsFDH-WT was also performed according to the manufacturer’s protocol (Qiagen). In order to calculate the molar absorption coefficients of TsFDH-WT and TsFDH-V13pAzF, the molar absorbance of AzF at 280 nm was measured by NanoDrop Spectrometer (Thermo Scientific, Wilmington, DE, USA), and found to be 2620 M^−1^cm^−1^. The molar absorption coefficients, ε_280_ (M^−1^cm^−1^), of TsFDH-WT and TsFDH-V13pAzF were calculated using the following equation, and determined to be 62,340 M^−1^cm^−1^ and 64,960 M^−1^cm^−1^, respectively [30]:

$${\text{ε}}_{280}=\left(5500\times {\text{n}}_{\text{Trp}}\right)+\left(1490\times {\text{n}}_{\text{Tyr}}\right)+\left(2620\times {\text{n}}_{\text{AzF}}\right)$$

The concentrations of purified TsFDH-WT and TsFDH-V13pAzF were measured using the Beer-Lambert Law [31]. The purified TsFDH-WT and TsFDH-V13pAzF (0.5 mg/mL) were subjected to trypsin digestion at 37 °C overnight, and then desalted on a ZipTip C18 according to the manufacturer’s protocol (Millipore). The tryptic digests were subjected to MALDI-TOF mass spectrometry (MS) as described previously except TsFDH variants instead of mDHFR variants [14].

### 3.4. Site-Specific Conjugation of Dye or Rh-Based Electron Mediator to TsFDH-V13pAzF

TsFDH-WT and TsFDH-V13pAzF (30 µM) in 50 mM HEPES (pH 8.2)/0.2 M NaCl were incubated with DBCO-PEG_4_-carboxyrhodamine (100 µM) at RT for 2 h, respectively. The reaction mixtures were subjected to SDS-PAGE, and then in-gel fluorescence (λ_ex_ = 480 nm; emission above 510 nm) was analyzed using a BioSpectrum imaging system (UVP, Upland, CA, USA). TsFDH-V13pAzF at 30 µM in 50 mM HEPES (pH 8.2)/0.2 M NaCl was reacted with DBCO-PEG_4_-DBCO at 90 µM at RT for 6 h. After desalting on a PD-10 column, RhM-azide at 300 µM was added to TsFDH-V13pAzF-DBCO-PEG_4_-DBCO conjugate at 30 µM and incubated at RT for 2 h to generate the TsFDH-RhM conjugate. Success of the RhM conjugation was confirmed by MALDI-TOF MS.

### 3.5. Functional Assay of the TsFDH-RhM Conjugate

The TsFDH-RhM conjugate (40 nM) was subjected to the enzyme activity assay by incubation with the assay buffer consisting of 40 mM formate and 2 mM NAD^+^ in 20 mM potassium phosphate/0.1M NaCl buffered at pH 8. As a positive and negative control, TsFDH-WT and RhM were incubated with the assay buffer, separately. The activity was measured by monitoring increase in absorbance at 340 nm. Photoinduced NADH generation by the conjugate or free RhM was investigated as described previously with some modifications in the reagent concentrations [25,26]. The reagent concentrations were adjusted to yield robust absorbance changes made by the RhM conjugated to TsFDH upon white light illumination. In brief, a reaction mixture consisting of either the conjugate (40 µM) or TsFDH (40 µM)/free RhM (40 µM), TEOA (5% v/v), eosin Y (40 µM), and NAD^+^ (800 µM) in 20 mM potassium phosphate/0.1 M NaCl buffered at pH 8 was illuminated by white light (5000K, 8W, and 265 lumens) without a cut-off filter. The samples were taken every 10 min and the absorbance was measured at 340 nm.

## 4. Conclusions

The artificial photosynthesis system investigated in this study consists of a sacrificial electron donor, dye-photosensitizer, cofactor, Rh-based electron mediator, and TsFDH. In particular, cofactors are key molecules in the cycle where they are oxidized by TsFDH, but photocatalytically regenerated. In order to facilitate cofactor oxidation and reduction, we exploited the conjugation of Rh-based electron mediator to a permissive site of TsFDH. First genetically engineered *E. coli* cells were used to incorporate pAzF into a permissive site (V13) of TsFDH while retaining full enzymatic activity. Second, bioorthogonal SPAAC was employed to conjugate RhM-azide to pAzF introduced to TsFDH in a site-specific manner. By carefully choosing a conjugation site, the resulting TsFDH-RhM conjugate retained the cofactor regenerating capacity and enzymatic activity on conversion of formate to CO_2_. We are currently working on engineering TsFDH to enhance its catalytic efficiencyas an important part of an artificial photosynthesis system. Although these results are preliminary, they represent a step forward in constructing an efficient artificial photosynthesis system to generate organic compounds.
